# Comparative transcriptomic profiling of the two-stage response of rice to *Xanthomonas oryzae* pv. *oryzicola* interaction with two different pathogenic strains

**DOI:** 10.1186/s12870-024-05060-1

**Published:** 2024-04-29

**Authors:** Yunya Bi, Yue Yu, Shuaige Mao, Tao Wu, Tao Wang, Ying Zhou, Kabin Xie, Hua Zhang, Li Liu, Zhaohui Chu

**Affiliations:** 1 State Key Laboratory of Hybrid Rice, Hubei Hongshan Laboratory, College of Life Sciences, Wuhan University, Wuhan, 430072 China; 2 State Key Laboratory of Biocatalysis and Enzyme Engineering, School of Life Sciences, Hubei University, Wuhan, 430062 China; 3 State Key Laboratory of Wheat Breeding, College of Agronomy, Shandong Agricultural University, Taian, 271018 China; 4 College of Life Sciences and Health, Wuhan University of Science and Technology, Wuhan, 430072 China; 5 Hubei Key Laboratory of Plant Pathology, Huazhong Agricultural University, Wuhan, 430070 China; 6 Tancheng Jinghua Seed Co., LTD, Linyi, Shandong 276100 China

**Keywords:** Rice, Bacterial leaf streak, Defense-responsive, Differentially expressed genes, Transcriptome profiling, *Xanthomonas oryzae* pv. *oryzicola*

## Abstract

**Background:**

Two-tiered plant immune responses involve cross-talk among defense-responsive (*DR*) genes involved in pathogen-associated molecular pattern (PAMP)-triggered immunity (PTI), effector-triggered immunity (ETI) and effector-triggered susceptibility (ETS). Bacterial leaf streak (BLS), caused by *Xanthomonas oryzae* pv. *oryzicola* (Xoc) is an important bacterial disease that causes serious threats to rice yield and quality. Transcriptomic profiling provides an effective approach for the comprehensive and large-scale detection of *DR* genes that participate in the interactions between rice and Xoc.

**Results:**

In this study, we used RNA-seq to analyze the differentially expressed genes (DEGs) in susceptible rice after inoculation with two naturally pathogenic Xoc strains, a hypervirulent strain, HGA4, and a relatively hypovirulent strain, RS105. First, bacterial growth curve and biomass quantification revealed that differential growth occurred beginning at 1 day post inoculation (dpi) and became more significant at 3 dpi. Additionally, we analyzed the DEGs at 12 h and 3 days post inoculation with two strains, representing the *DR* genes involved in the PTI and ETI/ETS responses, respectively. Gene Ontology (GO) functional and Kyoto Encyclopedia of Genes and Genomes (KEGG) pathway analyses were performed on the common DEGs, which included 4380 upregulated and 4019 downregulated genes and 930 upregulated and 1383 downregulated genes identified for the two strains at 12 h post inoculation (hpi) and 3 dpi, respectively. Compared to those at 12 hpi, at 3 dpi the number of common DEGs decreased, while the degree of differential expression was intensified. In addition, more disease-related GO pathways were enriched, and more transcription activator-like effector (TALE) putative target genes were upregulated in plants inoculated with HGA4 than in those inoculated with RS105 at 3 dpi. Then, four *DRs* were randomly selected for the BLS resistance assay. We found that *CDP3.10*, LOC_Os11g03820, and OsDSR2 positively regulated rice resistance to Xoc, while *OsSPX3* negatively regulated rice resistance.

**Conclusions:**

By using an enrichment method for RNA-seq, we identified a group of DEGs related to the two stages of response to the Xoc strain, which included four functionally identified *DR* genes.

**Supplementary Information:**

The online version contains supplementary material available at 10.1186/s12870-024-05060-1.

## Background

After thousands of years of evolution, pathogenic microorganisms and plants have experienced a complex and sophisticated ‘arms race’ of attack and defense [1]. When invaded by phytopathogens, plants deploy hundreds of receptor-like kinases (RLKs) and receptor-like proteins (RLPs) as pattern recognition receptors (PRRs) located on the surface of the cell membrane to quickly recognize pathogen-associated molecular patterns (PAMPs) and trigger PAMPs-triggered immunity (PTIs) to prevent further colonization and invasion [2]. To combat PTI, pathogens have evolved to secrete highly variable effectors to inhibit PTI, which is called effector-triggered susceptibility (ETS) [3]. Therefore, plants recognize pathogen effector proteins mostly through intracellular nucleotide-binding domains and leucine-rich repeat receptors (NLRs) to activate the effector-triggered immune (ETI) response and generate a hypersensitive response (HR) at the infection site to prevent further pathogen invasion [4]. Pathogens might then evolve novel effectors to suppress ETI and generate ETS. Generally, PTI and ETI use different receptors of PRRs and NLRs for signal perception; however, they share several well-documented downstream signaling pathways, such as the activation of mitogen-activated protein kinase (MAPK) cascades, reactive oxygen species (ROS) bursts, hormone signaling transduction, and transcriptional reprogramming of defense-responsive (*DR*) genes [5, 6]. The specific effectors involved in the regulation of ETI or ETS have been explored. However, global transcription profiling of *DR* genes involved in ETI/ETS has rarely been performed.

Bacterial leaf streak of rice (BLS), caused by *Xanthomonas oryzae* pv. *oryzicola* (Xoc), is an important bacterial disease in rice [7]. In particular, this phytosanitary disease severely threatens rice yield and quality and seed production in China. Xoc can secrete a variety of effector proteins with virulence, nontoxic functions or both. Among them, transcriptional activator-like effectors (TALEs) are mainly injected into host cells via the type III secretion system (T3SS), thus overcoming the PTI immune response of plants and promoting bacterial growth [8]. TALEs are trapped by effector-binding elements (EBEs) in the promoter region of host susceptibility (*S*) or resistance (*R*) genes through repeat-variable diresidues (RVDs) to activate the transcription of those genes to facilitate bacterial proliferation or defense [9, 10]. Globally, the exploration of TALE-rice interactions has been dependent mainly on revealing putative target genes by predicting EBEs with RVDs of TALEs [9]. However, there are several TALEs with no identical target genes, which limits the ability to reveal the mechanism of ETI/ETS in rice-Xoc interactions [11].

The expression of the *DR* gene is rapidly influenced by pathogens and elicitors. Modification of *DR* gene expression can improve rice resistance to Xoc. In addition to singleton *DR* genes, some *DR* gene families, such as *OsWRKY45-1* and *OsWRKY45-2* [12], the polygalacturonase inhibiting protein genes *OsPGIP1* and *OsPGIP4* [13], and the salicylic acid (SA) metabolic enzyme family *OsF3H03g*, *OsF3H04g*, and *OsS3H* [14–16], have also been reported to be involved in BLS resistance. Some transcription modules, such as OsHsfB4d-OsHsp18.0-CI [17] and OsASR6-OsCIPK15 [18], are also involved in BLS resistance. Nonetheless, the defense mechanisms against BLS, particularly the intricacies of the signaling networks associated with these *DR* genes, remain unclear. Because preventing the infection cycle as early as possible is beneficial for preventing the disease, most of the identified *DR* genes are upregulated in the early stages of infection. Therefore, there is a crucial need to globally identify more *DR* genes, especially those that respond at later stages of pathogen attack.

Since the completion of rice genome sequencing projects, large-scale, high-throughput techniques for gene function analysis have emerged as a critical objective. Transcriptomic sequencing has been developed as an effective method to discover the regulatory and signaling networks of rice *DR* genes. However, large numbers of differentially expressed genes (DEGs) or *DR* candidates still make functional assays of each gene difficult. Comparative transcriptomics strategies seek to enhance our understanding of DEGs by analyzing transcriptomic variations across different samples within the same species or among various species [19]. Identification of *DR* genes via transcriptomic profiling of the rice variety Hongyou-4 via inoculation with compatible and incompatible Xoc strains revealed 8 DEGs, including those encoding transcription factors (TFs), *R* gene analogs and the *S* gene of *OsSWEET13* [20]. Tang et al. reported the mining of five *DR* genes via the inoculation of a rice near-isogenic line of NLS-*bls2* after inoculation with a wild-type Xoc strain and a TALE-knockout Xoc strain, which conferred compatible and incompatible interactions, respectively, on rice [21]. Meng et al. identified potential disease resistance genes by comparing the transcriptomes of powdery mildew-resistant and powdery mildew-susceptible cucumber materials [22]. Cao et al. identified 388 potential key *DR* genes for banded leaf and sheath blight (BLSB) resistance by analyzing the transcriptomes of maize plants infected with a hypovirulent strain or a hypervirulent strain of *Rhizoctonia solani* for 3 or 5 days via transcriptomic deep sequencing [23]. Comparative transcriptomics analysis is used to reveal the expression patterns of conserved genes and identify homologs that perform the same function in different organisms, highlight genes that are critical to biological processes, and study gene evolution from the perspective of gene expression. Overall, comparative transcriptome analysis will help to identify *DR* genes and provide us with a better understanding of the immune response related to resistance and susceptibility.

Previously, HGA4 was demonstrated to be more virulent than RS105 in eight japonica rice varieties and six indica rice varieties [14]. Compared with RS105, HGA4 contain four more TALEs, including Tal2b, Tal2c, Tal2d and Tal2e, which contribute to the major increase in virulence [14]. Among the four TALEs, Tal2b and Tal2c target the *S* genes of *OsF3H03g* and *OsF3H04g*, respectively, by binding to the EBEs in their promoters [14, 15]. However, there are no predicted targets for Tal2d or Tal2e in rice [14]. In this study, we aimed to enrich the *DR* genes for rice-Xoc interactions by performing comparative transcription profiling of two naturally different virulent strains, RS105 and HGA4, which are defined as a hypovirulent strain and a hypervirulent strain, respectively. We investigated the DEGs and their transcriptional changes after inoculation with each strain for 12 h (h) and 3 days (d). Bioinformatic analysis and expression validation with quantitative RT‒PCR were performed with the enriched *DR* genes, especially for those DEGs associated with ETI/ETS function at 3 days post inoculation (dpi). The functions of four candidate *DR* genes in regulating BLS resistance in transgenic rice lines were investigated.

## Materials and methods

### Plant materials and growth conditions

In addition to special instructions, the rice materials used in this study were *Oryza sativa* L. spp. *japonica* rice ZH11. *OsDSR2-*overexpressing and RNAi lines were derived from ZH11 and kindly provided by Professor Liang Chen at Xiamen University [24]. The *LOC_Os11g03820* mutant in the KitaaKe background was generated from a published reference [25], and seeds of the other two *oscdp3.10* and *osspx3 mutants* were purchased from Wimibio (http://www.wimibio.com/, BG100108C10 and BG100147F12, respectively). The rice plants were grown in a phytotron of a plant growth breeding system (PGBS, Wuhan Greenfafa Institute of Novel Genechip R&D Co., Ltd., Wuhan, China) at a temperature of 26 ± 2 °C, 85-100% humidity, and a photoperiod of 14 h.

### Bacterial inoculation, biomass quantification and lesion statistics

The Xoc strains RS105 and HGA4 were grown on PSA media (10 g·L^− 1^ polypeptone, 1 g·L^− 1^ glutamic acid, 10 g·L^− 1^ sucrose and 15 g·L^− 1^ agar, pH = 7.0) at 28 °C for 2–3 d and then suspended in ddH_2_O to an adjusted OD_600_ = 0.5. Four-week-old plants were inoculated with HGA4 and RS105 on extended leaves using a 2.5 mL needle-less syringe [14]. Five centimeters long leaves flanking the inoculation site were collected for the experiments. The leaves inoculated for 0.5, 1, 2 and 3 d were used to generate the bacterial growth curve as previously reported [26]. DNA was extracted and used as a template for relative quantitative PCR with OsUBQ and XOC0105 [27] to quantify the relative amount of DNA of rice and bacteria, respectively. The lesion length was measured at 14 dpi.

### RNA-seq data analysis

ZH11 leaves inoculated with HGA4 and RS105 at 12 h and 3 d were collected for RNA-seq, and uninoculated leaves were used as controls. As previously reported [16], total RNA was isolated from rice leaves using TRI reagent (Sigma‒Aldrich, USA). Triplicate RNA samples were sequenced with BGISEQ-500 by the Beijing Genomic Institution (Shenzhen, China). The preliminary analysis of the data was performed according to the BGI standard operating procedure (http://bgitechsolutions.com/sequencing/45). Genes with *p* ≤ 0.05 and | Log_2_ (fold change) | ≥ 1 were selected as DEGs. GO enrichment analysis was performed using AgriGO (https://bioinfo.cau.edu.cn/agriGO, accessed on 11 November 2022) to clarify their main biological functions. The software KOBAS (http://kobas.cbi.pku.edu.cn/genelist, accessed on 23 July 2023) was used to determine the abundance of DEGs in the KEGG pathway and analyze the genes and fluxes related to plant disease resistance. The transcriptome dataset has been deposited in the NCBI Sequence Read Archive Database (http://trace.ncbi.nlm.nih.gov/Traces/sra) under accession number PRJNA1033788.

### Detection of relative gene expression

The concentration and the A260/A280 ratio of the extracted total RNA were measured using a spectrophotometer (ND-100 F, MIULAB, Hangzhou, China). RNA reverse transcription was performed using EasyScript® One-Step gDNA Removal and cDNA Synthesis SuperMix (TransGene, Beijing, China). The cDNA products were subjected to qRT‒PCR using PerfectStart® Green qPCR SuperMix (TransGene) on a CFX Connect instrument (Bio-Rad, Hercules, CA, USA). The relative quantitative 2^−ΔΔCT method^ was used to compare and analyze gene expression [28]. The primers used are shown in Supplemental Table 1.

### Positive selection of transgenic plants

Rice leaf DNA was extracted with a Plant Genomic DNA Kit (CWBIO, Beijing, China). The *LOC_Os11g03820* mutant, *oscdp3.10* and *osspx3* were generated by CRISPR/Cas9. Primers flanking the target site were designed and are listed in Supplemental Table 1. The amplified DNA was purified from gels and sequenced with AuGCT (http://www.augct.com/). The sequence was aligned with that of the wild type to identify the homozygous mutant plants. Positive plants of the OsDSR2-overexpressing and RNAi lines were directly identified via PCR using specific primers as previously reported [24].

### Statistical analysis

All data analysis was performed in Microsoft Excel and GraphPad Prism 9 (GraphPad Software, La Jolla, CA, USA). Data are presented as means ± SD (unless otherwise noted). One-way ANOVA with Dunnett’s multiple comparisons test or two-way ANOVA with Šídák multiple comparisons test was performed to compare multiple groups.

## Results

### Determination of the relative bacterial population of HGA4 and RS105 in rice

**Fig. 1:**
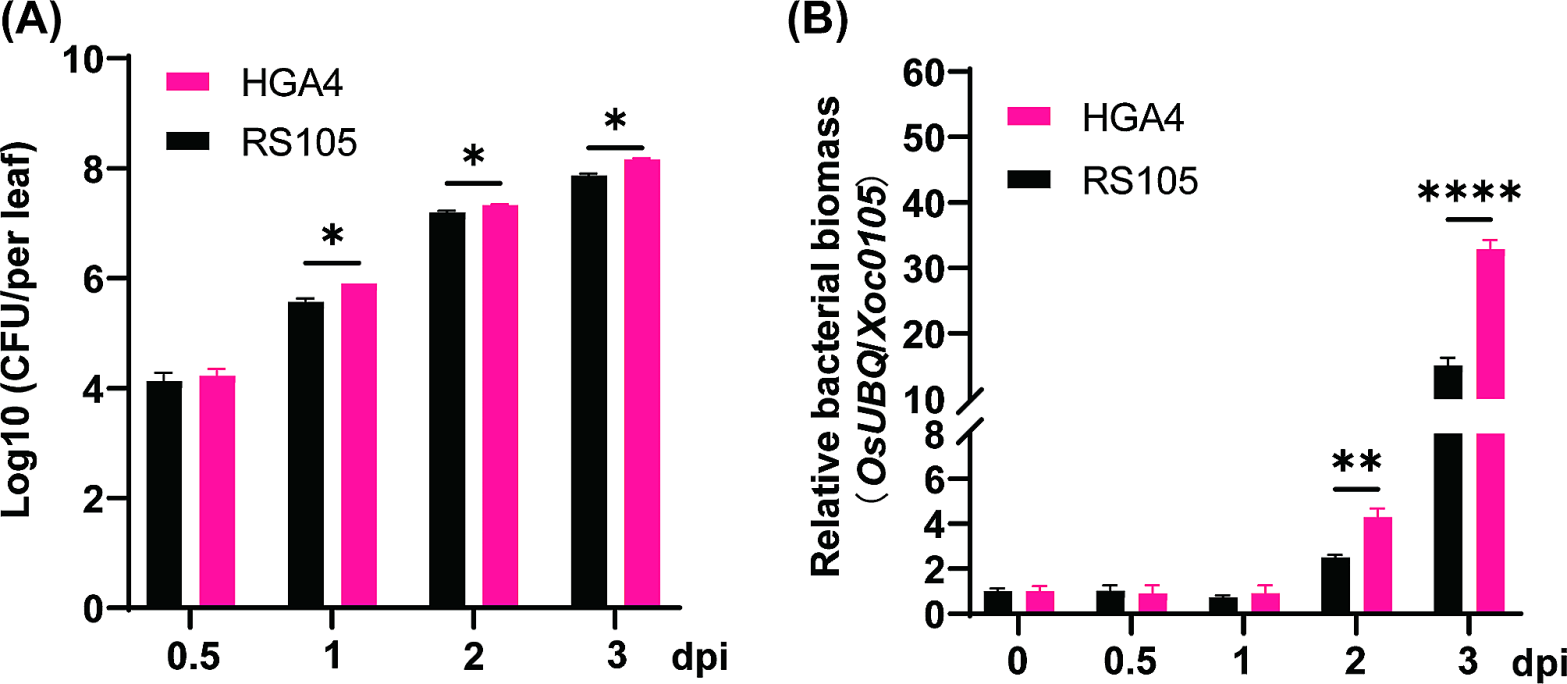
Time-point investigation of the bacterial population inoculated with HGA4 and RS105. **(A)** The bacterial population in ZH11 at 0.5, 1, 2 and 3 dpi with HGA4 and RS105. The error bars represent the means ± SDs (*n* = 3). * indicates a significant difference between HGA4 and RS105 (* *P* ≤ 0.05, two-way ANOVA). **(B)** Relative bacterial biomass of HGA4 and RS105 at 0, 0.5, 1, 2, and 3 dpi in ZH11. The error bars represent the means ± SDs (*n* = 3). * indicates a significant difference between HGA4 and RS105 (**P ≤ 0.01, **** *P* ≤ 0.0001, two-way ANOVA with Šídák multiple comparisons test)

A previous study indicated that HGA4 is more virulent than RS105 [13, 14]. To ascertain the time point marking the divergence in pathogenicity between HGA4 and RS105, we inoculated the *japonica* cultivar ‘Zhonghua 11’ (ZH11) with two distinct strains. The biomass was measured by using bacterial growth count and qPCR quantification. We found that there was no significant difference between the two Xoc strains at 12 h (0.5 d) post inoculation (hpi), while a significant difference was observed at 1, 2, and 3 dpi (Fig. 1A). Furthermore, qPCR analysis of bacterial biomass also validated the bacterial growth curve results (Fig. 1B). To accurately investigate the difference in gene expression between HGA4 and RS105 at the early and later stages of inoculation, respectively, we selected leaves at 12 hpi and 3 dpi for RNA-seq to identify *DR* genes.

### General analysis of RNA-seq results

To investigate the differentially expressed genes (DEGs) in rice after inoculation with two Xoc strains, HGA4 and RS105, the total RNA of ZH11 leaves was subjected to RNA-seq at 12 hpi and 3 dpi, respectively; noninoculated leaves were used as controls. We sequenced a total of 15 libraries for all five samples (three biological replicates each for 12 h_HGA4, 3 d_HGA4, 12 h_RS105, 3 d_RS105 and the control), and an average of 21.65 million clean reads were obtained. The average alignment rate of the clean reads to the reference genome was 96.35% (Table S2). The expression of genes after inoculation with HGA4 and RS105 was compared with that of the control. DEGs were identified based on a threshold of | Log_2_ (fold change) | ≥ 1 and *P* ≤ 0.05.

### Analysis of DEGs at 12 hpi

**Fig. 2:**
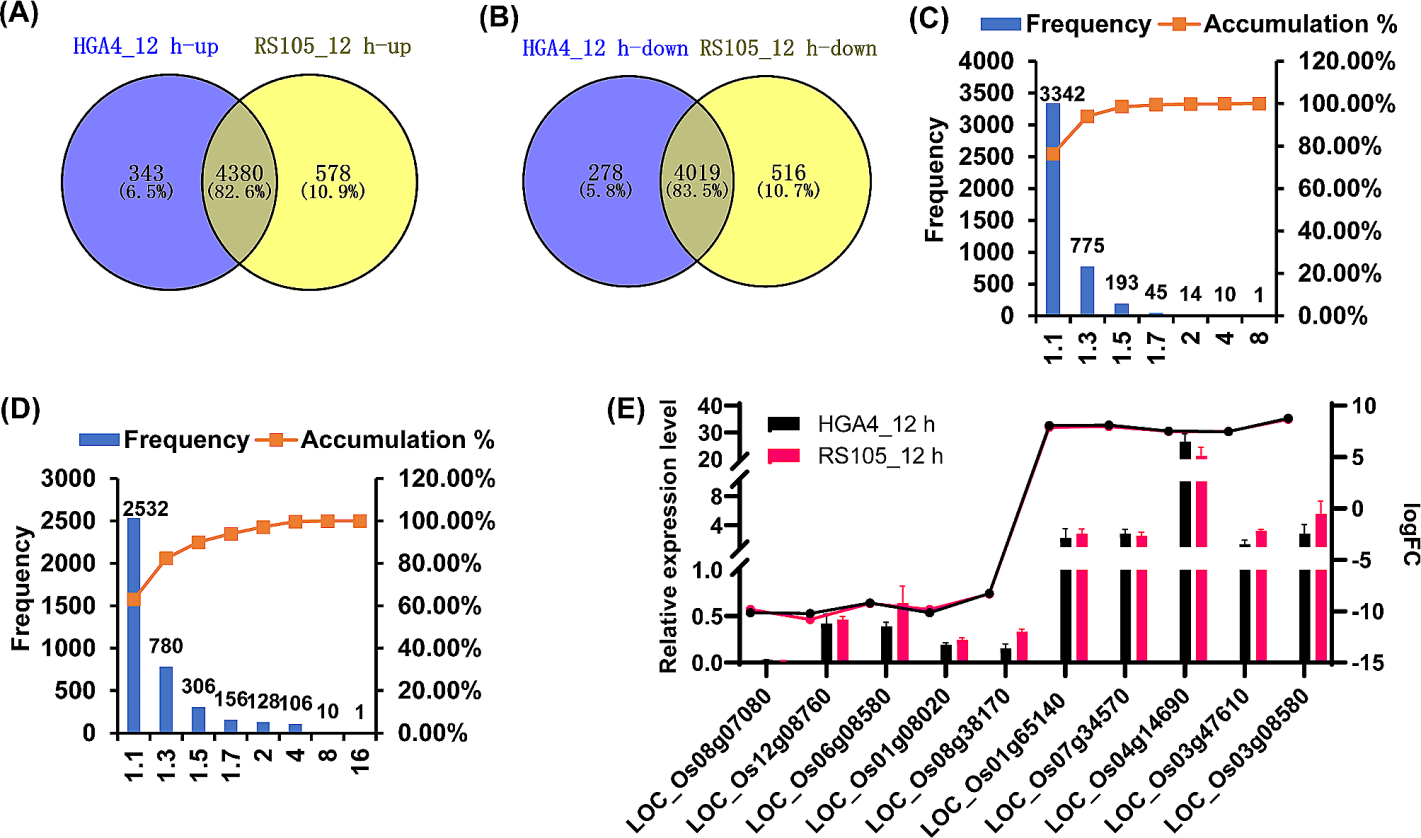
Analysis of DEGs at 12 h post inoculation (hpi) with HGA4 and RS105. **(A, B)** Venn diagram of upregulated **(A)** and downregulated DEGs **(B)**. **(C, D)** Histogram of the ratio of upregulated common DEGs **(C)** and downregulated common DEGs (**D**) at 12 hpi with HGA4 and RS105. **(E)** Validation of the common DEGs by qRT‒PCR. The line chart represents the Log2 (fold change) value of the transcriptome, and the histogram represents the qRT‒PCR results

A total of 9020 and 9493 DEGs were identified at 12 hpi with HGA4 and RS105, respectively. Among these DEGs, 4,380 upregulated and 4,019 downregulated DEGs were common to both HGA4 and RS105, representing 82.6% and 83.5% of their identified DEGs, respectively (Fig. 2A, B). Furthermore, a comparison of the relative expression levels of the common DEGs revealed that 98.4% of the upregulated and 90.02% of the downregulated common DEGs exhibited less than a 1.5-fold difference in expression between HGA4 or RS105 inoculation (Fig. 2C, D). These results suggest that two different virulent strains affect the expression of host genes similarly at both the gene number and expression level at the early stage of 12 hpi.

To understand the functions of the common DEGs, we annotated these genes (Table S3), which included genes encoding transcription factors (MYB, WRKY, ERF, and EREBP), protein kinase-related proteins (WAKs and SAPKs) and antibody protein-related genes (disease resistance, cytochrome P450, regulation of response to stimulus, LRRs, and natural resistance-associated macrophage protein) [21]. In addition, we identified five genes closely related to BLS resistance based on previous reports [12, 25, 29–31], including *OsPGIP1* (*LOC_Os05g01380*), the phytosulfokine receptor gene Os*PSKR1* (*LOC_Os02g41890*), the resistance gene *OsBLS1* (*LOC_Os06g06090*), the bHLH transcription activator regulator gene *OsbHLH6* (*LOC_Os04g23550*), and the sulfate transporter gene *OsSULTR3;6* (*LOC_Os01g52130*). We also performed Gene Ontology (GO) analysis for these common DEGs. For upregulated common DEGs, three prominent biological process-related terms were “translation (GO:0006412)”, “cellular protein metabolic process (GO:0044267)” and “gene expression (GO:0010467)” (Fig. S1A). Some functional categories related to the defense response were also enriched, such as “response to oxidative stress (GO:0006979)”, “response to stress (GO:0006950)”, “cellular response to stimulus (GO:0050896)”, and “response to chemical stimulus (GO:0042221)”, among others (Table S4). GO analysis revealed that downregulated DEGs were mainly enriched in a large number of biological regulation processes (Fig. S1B), such as “regulation of biological process (GO:0050789)”, “regulation of transcription (GO:0045449)”, etc. (Table S5). We randomly selected each of the 5 common upregulated and downregulated DEGs for validation by using qRT‒PCR, and the results were consistent with the RNA‒seq results (Fig. 2E).

Overall, the above results showed that the expression of a large number of genes related to the immune response was altered, which needs to be further investigated via functional assays.

### Analysis of DEGs at 3 dpi

**Fig. 3:**
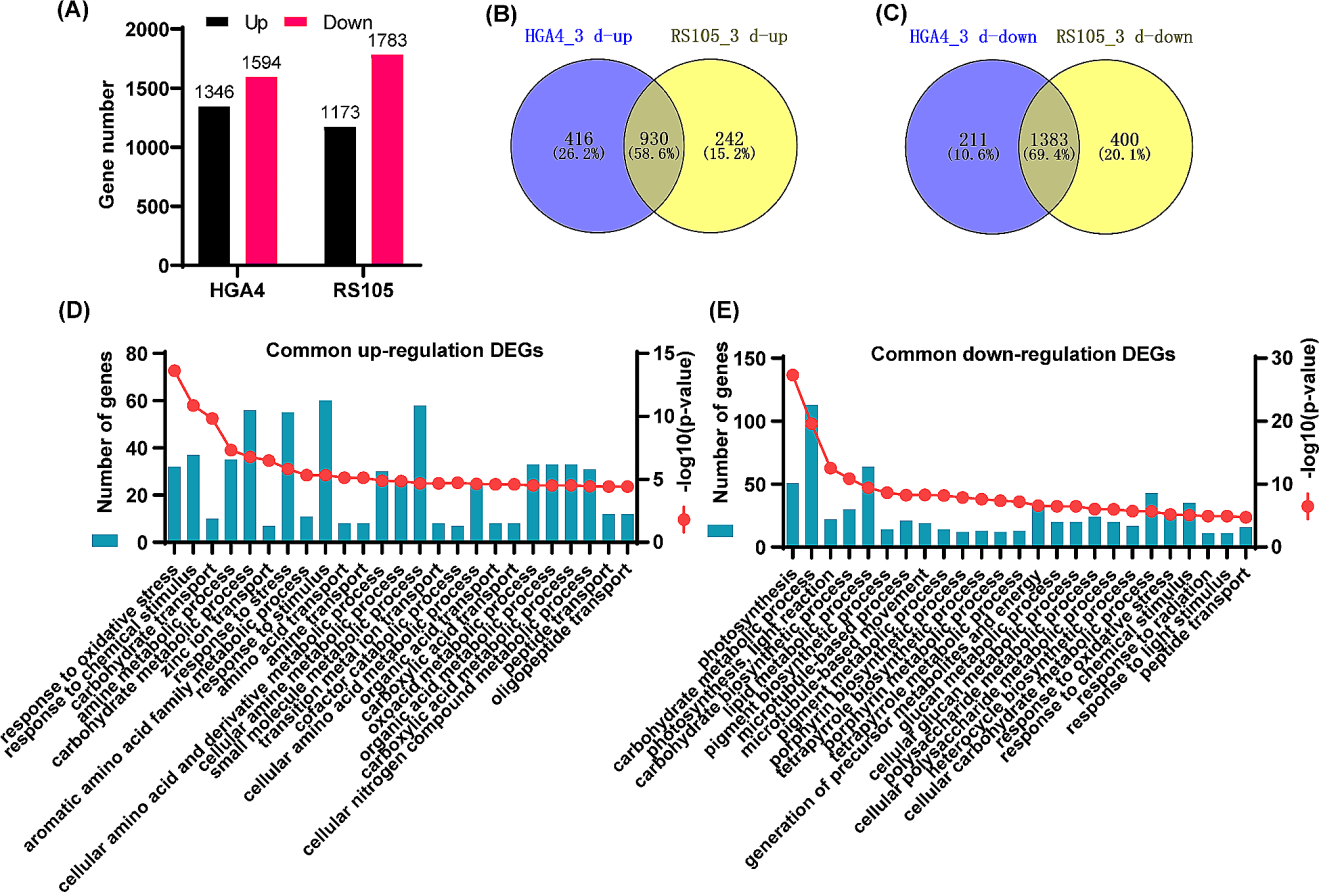
Identification of the common DEGs at 3 days post inoculation (dpi) with HGA4 and RS105. **(A)** Number of DEGs in plants inoculated with HGA4 and RS105 at 3 dpi. **(B, C)** Venn diagram of upregulated **(B)** and downregulated DEGs **(C).** Biological process analysis of the common DEGs between the upregulated **(D)** and downregulated **(E)** DEGs

A total of 1346 upregulated and 1594 downregulated DEGs and 1173 upregulated and 1783 downregulated DEGs were obtained at 3 dpi in ZH11 inoculated with HGA4 and RS105, respectively (Fig. 3A). A Venn diagram revealed that 58.6% of the upregulated DEGs and 69.4% of the downregulated DEGs were common DEGs after inoculation with HGA4 and RS105 (Fig. 3B, C). We further performed a GO analysis for these common DEGs. The upregulated common DEGs were significantly enriched in GO terms such as “response to oxidative stress (GO:0006979)”, “response to chemical stimulus (GO:0042221)”, “response to stress (GO:0006950)”, and “response to stimulus (GO:0050896)” (Fig. 3D; Table S6). Two prominent biological process-related terms of the downregulated DEGs were “photosynthesis” and “carbohydrate metabolic process” (Fig. 3E; Table S7). Furthermore, an analysis of the relative expression levels revealed that 21.87% of the upregulated and 20.25% of the downregulated common DEGs exhibited a variation greater than 1.5-fold between the inoculations with HGA4 and RS105, indicating a significant differential response to these two treatments associated with pathogenicity (Fig. S2).

There are 28 and 24 TALEs in HGA4 and RS105, respectively, which mediate the activation of *S* or *R* genes by binding to their promoters in rice [14]. To date, a total of 13 TALEs have been predicted to target 19 rice genes [32, 33]. Here, we screened all potential TALE target genes in ZH11 to evaluate the differences in their expression patterns between HGA4 and RS105. According to the RNA-seq data, 15 and 10 potential target genes were upregulated at 3 dpi with HGA4 and RS105, respectively. Furthermore, we observed that all 10 common potential target genes, including the previously reported *LOC_Os06g46500* of Tal2g (BLS256) and *LOC_Os09g29100* of Tal7 (RS105), were more strongly induced upon inoculation with HGA4 than upon inoculation with RS105 (Table 1). Notably, two other identified HGA4-containing TALE target genes, *OsF3H03g* of Tal2b and *OsF3H04g* of Tal2c, were also observed among the HGA4-specific DEGs (Table 1). We selected four genes for qRT-PCR to detect changes in their expression levels. The results of qRT-PCR also showed that there were significant differences between HGA4 and RS105 at 3 dpi (Fig S3).

**Table 1:** The expression level of putative target genes of TALEs

GENE ID	TALE^a^			Log2 (fold change)			
	BLS256	HGA4	RS105	HGA4 _12 h	RS105 _12 h	HGA4 _3 d	RS105 _3 d
LOC_Os07g06970 (*OsHEN1*)	Tal1c	Tal13b	Tal12b	1.94	1.47	2.77	N/A^b^
LOC_Os02g43760	Tal2a	Tal3	Tal5a	0.44	0.41	0.60	N/A
LOC_Os01g52130 (*OsSULTR3;6*)	Tal2g	Tal2g	Tal5d	6.14	5.00	8.75	7.83
LOC_Os06g46500				1.94	0.93	5.33	4.16
LOC_Os02g34970	Ta3b	Tal5	Tal6b	N/A	N/A	4.50	3.51
LOC_Os05g27590				N/A	N/A	3.78	3.34
LOC_Os07g36430				N/A	N/A	4.91	4.29
LOC_Os02g47660	Tal3c	Tal6	Tal6c	N/A	-0.50	2.00	1.40
LOC_Os03g07540				5.54	4.15	6.29	5.96
LOC_Os03g37840	Tal4a	Tal10a	Tal9a	-1.61	-1.55	0.63	0.56
LOC_Os09g32100	Tal4b	Tal10b	Tal9b	-1.72	-2.34	2.61	1.99
LOC_Os06g37080	Tal4c	Tal10c	Tal9c	4.10	3.78	5.82	4.95
LOC_Os02g15290	Tal5a	Tal9	Tal8a	-1.75	-2.82	0.58	N/A
LOC_Os09g29100	Tal6	Tal7	Tal7	3.04	2.58	3.75	2.48
LOC_Os12g42970				N/A	N/A	1.00	0.59
LOC_Os01g31220				0.31	N/A	1.23	0.65
LOC_Os01g51040	Tal9b	Tal18b	Tal4b	-3.48	-3.41	0.76	N/A
LOC_Os03g03034 (*OsF3H03g*)	Tal2c	Tal2b		0.74	-0.87	3.30	0.80
LOC_Os04g49194 (*OsF3H04g*)	Tal2d	Tal2c		-1.16	-2.08	4.03	-0.96

^a^TALE represents the name of homologous in different *Xanthomonas oryzae* pv. *oryzicola* races [13, 32, 33]

^b^N/A represented that no transcript was detected

### Comparison of common DEGs at 12 hpi and 3 dpi

**Fig. 4:**
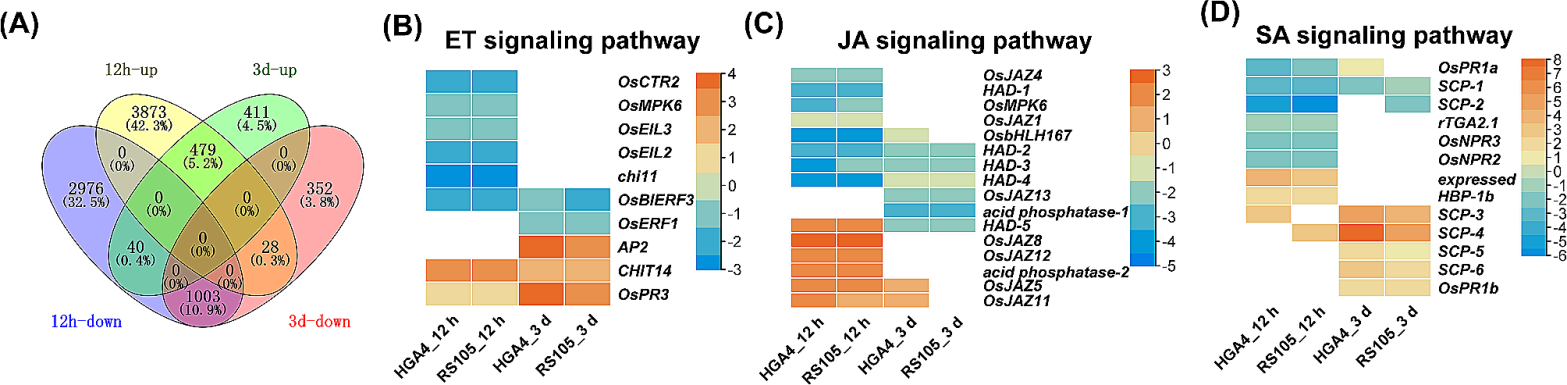
Assessment of the cross-common DEGs at 12 hpi and 3 dpi. **(A)** Venn diagram of the cross common DEGs. (B-D) Heatmap of DEGs in the ET signaling pathway **(B)**, JA signaling pathway **(C)** and SA signaling pathway **(D).** HAD represents HAD superfamily phosphatase; SCP represents SCP-like extracellular protein

Compared with those at 12 hpi, the numbers of total and common DEGs were significantly lower for both the upregulated and downregulated plants at 3 dpi. In addition, the percentage of common DEGs among the total DEGs induced by HGA4 and RS105 decreased significantly at 3 dpi (upregulation decreased from 82.6 to 58.6%, downregulation decreased from 83.5 to 69.4%) (Figs. 2A and B and 3B and C). Compared with those common DEGs identified at 12 hpi, the differential expression levels of common DEGs of HGA4 and RS105 were more significant at 3 dpi (Fig. 2C and D, S2). GO analysis also revealed that disease resistance-related pathways were more significantly enriched at 3 dpi than at 12 hpi (Fig. 3D and E, S1). We further analyzed the common DEGs by combining the common DEGs at 12 hpi and 3 dpi. A total of 479 upregulated and 1003 downregulated DEGs were identified at both 12 hpi and 3 dpi, 411 upregulated and 352 downregulated DEGs were specifically identified at 3 dpi, and 3873 upregulated and 2976 downregulated DEGs were specifically identified at 12 hpi (Fig. 4A). Among the thirteen reported BLS *DR* genes, seven were upregulated at both 12 hpi and 3 dpi (Table 2). Four genes were downregulated at 12 hpi but were not detected or expressed without significant changes at 3 dpi. The expression of two genes increased at 3 dpi but did not significantly change at 12 hpi (Table 2). Among the common potential TALE target genes, four genes (*OsSULTR3;6*, *LOC_Os03g07540*, *LOC_Os06g37080* and *LOC_Os09g29100*) were upregulated at both 12 hpi and 3 dpi, five genes (*LOC_Os02g34970*, *LOC_Os05g27590*, *LOC_Os07g36430*, *LOC_Os02g47660* and *LOC_Os09g32100*) did not change or downregulated in expression at 12 hpi, and one gene (*LOC_Os06g46500*) was upregulated by HGA4 but was not significantly altered by RS105 at 12 hpi (Table 1). These results indicated that similar gene expression was activated in rice in response to invasion by different virulent Xoc strains at the early stage, but gene expression profiling differed at the later stage.

**Table 2:** Relative expression levels of DEGs with specific functions

MSU_Locus	Gene name	Log2 (fold change)			
		HGA4_12 h	RS105_12 h	HGA4_3 d	RS105_3 d
LOC _Os01g52130	*OsSULTR3;6*	6.14	5.00	8.75	7.83
LOC_Os05g01380	*OsPGIP1*	6.09	6.12	4.01	3.67
LOC_Os02g41510	*OsMYB30*	4.22	4.19	2.65	1.79
LOC_Os03g18850	*JIOsPR10*	4.03	4.13	4.55	4.42
LOC_Os07g15460	*OsNRAMP1*	3.58	3.80	1.88	2.16
LOC_Os02g41890	*OsPSKR1*	1.69	1.74	1.02	1.17
LOC_Os11g45740	*OsJAMyb*	1.64	1.58	1.87	1.37
LOC_Os06g06090	*BLS1*	-1.06	-1.19	NA^a^	NA
LOC_Os05g25770	*OsWRKY45*	-1.75	-2.41	-0.76	-0.56
LOC_Os03g16030	*OsHSP18.0-CI*	-2.67	-2.36	NA	NA
LOC_Os05g30250	*OsBGLU19*	-4.28	-3.91	-0.60	-0.48
LOC_Os11g02240	*OsCIPK15*	0.99	0.88	1.86	1.07
LOC_Os01g55940	*OsGH3-2*	-0.88	-0.50	1.17	1.28

^a^N/A represented that no transcrip was detected

Salicylic acid (SA), jasmonic acid (JA) and ethylene (ET) are central signaling molecules that coordinate plant defenses against microbial pathogens with different lifestyles [34, 35]. We also identified 39 DEGs enriched in the hormone signaling pathway and MAPK signaling pathway KEGG pathways (Fig. S4, Table S8). There were 10, 16 and 13 DEGs in the ET, JA and SA signaling pathways, respectively. By analyzing the expression patterns, we found that ten ET-responsive genes could be further classified into four categories. Five genes were downregulated at 12 hpi but not at 3 dpi, namely, *OsCTR2* (*LOC_Os02g32610*), the mitogen-activated protein kinase gene *OsMPK6* (*LOC_Os06g06090*), the ethylene signaling regulator genes *OsEIL3* (*LOC_Os09g31400*) and *OsEIL2* (*LOC_Os07g48630*), chitinase gene *Oschi11* (*LOC_Os03g04060*); one genes were downregulated at 3 dpi, namely, the ethylene response factor genes *OsERF1* (*LOC_Os04g46220*); and one genes were downregulated at both 12 hpi and 3 dpi, namely, *OsBIERF3* (*LOC_Os02g43790*); two genes were upregulated both at 12 hpi and 3 dpi, namely, the chitinase gene *OsPR3* (*LOC_Os06g51050*) and the chitinase family protein precursor gene *OsCHIT14* (*LOC_Os10g39680*) (Fig. 4B). In response to pathogen invasion, the JA signaling inhibitor genes *OsJAZ4* (*LOC_Os09g23660*) and *OsJAZ1* (*LOC_Os04g55920*) were downregulated at 12 hpi, while *OsJAZ13* (*LOC_Os10g25230*) was downregulated at 3 dpi, and *OsJAZ8* (*LOC_Os09g26780*), *OsJAZ12* (*LOC_Os10g25290*), *OsJAZ5* (*LOC_Os04g32480*), and *OsJAZ11* (*LOC_Os03g08320*) were upregulated at 12 hpi (Fig. 4C). OsNPR1 interacts with rice transcription factor OsrTGA2.1 (LOC_Os07g48820) and positively regulate rice resistance to Xoc, while silencing *OsrTGA2.1* increases rice resistance against bacterial pathogens [36, 37]. The SA signaling pathway-related gene *OsrTGA2.1* was downregulated at 12 hpi, and the NPR1-like genes *OsNPR2* (*LOC_Os01g56200*) and *OsNPR3* (*LOC_Os03g46440*) were also downregulated at 12 hpi (Fig. 4D).

### Verification of disease resistance for the identified DEGs

**Fig. 5:**
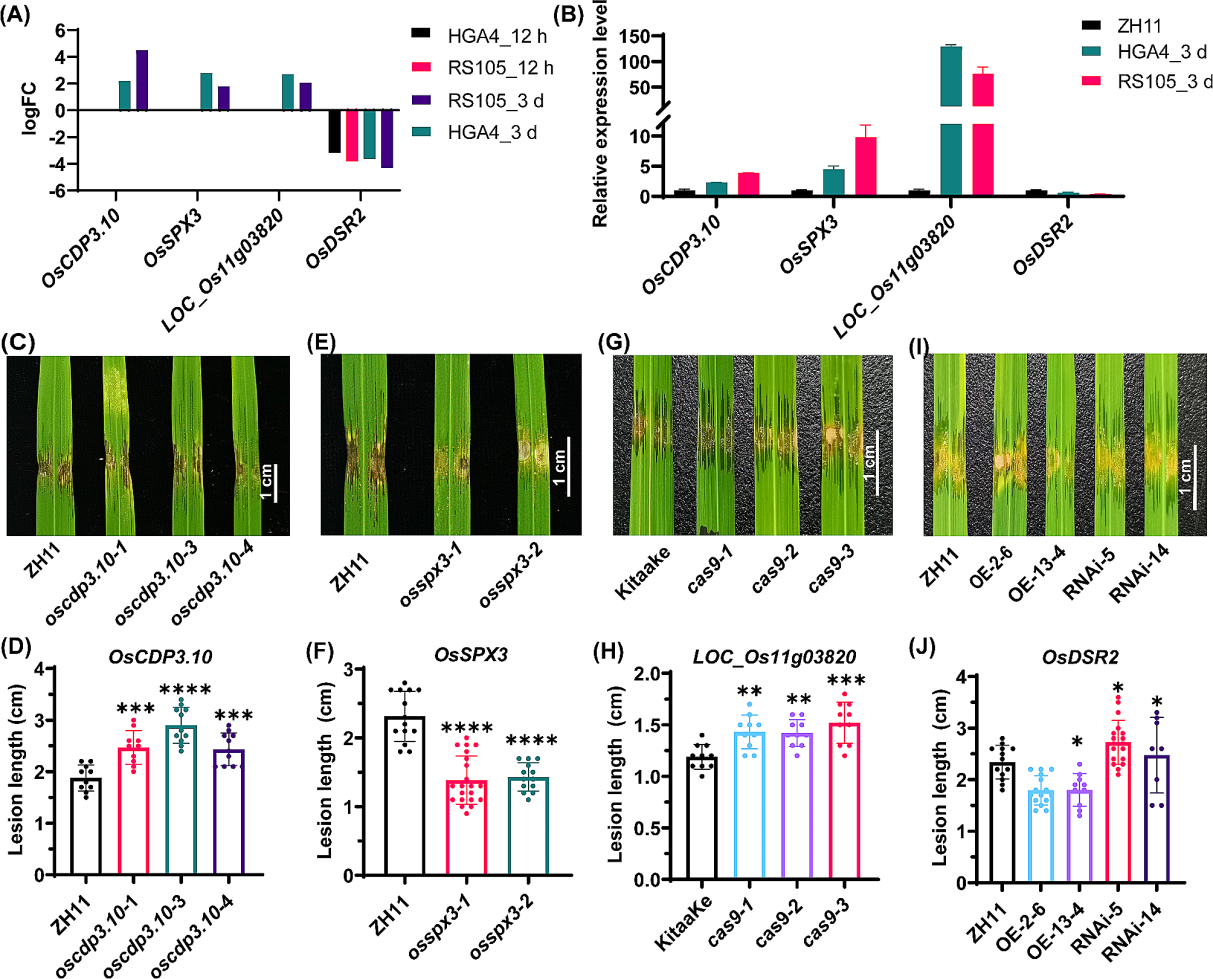
Disease resistance of the *OsCDP3.10*, *OsSPX3*, *LOC_Os11g03820* and *OsDSR2* transgenic rice lines. **(A, B)** Expression levels determined by RNA-seq **(A)** and qRT‒PCR (**B**). **(C, D)** Image of lesion expansions **(C)** and diagram of lesion lengths **(D)** for the *OsCDP3.10* gene-edited lines (*oscdp3.10-1*, *oscdp3.10-3* and *oscdp3.10-4*) and wild-type ZH11. The error bars represent the means ± SDs (*n* = 10). **(E, F)** Image of lesion expansions **(E)** and diagram of lesion lengths **(F)** for the *OsSPX3* gene-edited lines (*osspx3-1* and *osspx3-2*) and ZH11. The error bars represent the means ± SDs (*n* = 13). **(G, H)** Image of lesion expansions **(G)** and diagram of lesion lengths **(H)** for the *LOC_Os11g03820* gene-edited lines (*cas9-1*, *cas9-2* and *cas9-3*) and the wild-type Kitaate. The error bars represent the means ± SDs (*n* = 10). **(I, J)** Image of lesion expansions **(I)** and diagram of lesion lengths **(J)** for the *OsDSR2* transgenic lines (*OE-2-6*, *OE-13-4*, *RNAi-5* and *RNAi-14*) and ZH11. The error bars represent the means ± SDs (*n* ≥ 8). All plants were inoculated with Xoc HGA4, and the data were collected at 14 dpi. Scale bar = 1 cm. Asterisks represent significant differences between the gene-edited lines and wild-type plants (*P ≤ 0.05, ** *P* ≤ 0.01, *** *P* ≤ 0.001, **** *P* ≤ 0.0001, One-way ANOVA with Dunnett’s multiple comparisons test)

Fewer DEGs related to rice defense were more enriched at 3 dpi than at 12 hpi. These genes also exhibited increased enrichment of TALE-targeting *S* genes and their downstream components involved in the ETI/ETS response. We focused on the DEGs at 3 dpi and randomly selected four genes for which the mutants or transgenic seeds could be obtained (Fig. 5A, Table S9). These genes included *OsCDP3.10* (*LOC_Os03g57960*), which encodes a cupin domain protein [38]; *OsSPX3* (*LOC_Os10g25310*), which encodes an SPX family protein [39]; *LOC_Os11g03820*, which encodes an RLK family [25]; and *OsDSR2* (*LOC_Os01g62200*), which encodes a DUF966 stress-repressive protein [24]. Three genes, *OsCDP3.10, OsSPX3* and *LOC_Os11g03820*, were upregulated by both RS105 and HGA4 at 3 dpi but not at 12 hpi, whereas OsDSR2 was downregulated for RS105 and HGA4 at 12 hpi and 3 dpi (Fig. 5A and B). After inoculation with HGA4, the lesion lengths of the CRISPR/Cas9 lines *oscdp3.10-1* (2.47 ± 0.33 cm), *oscdp3.10-3 (*2.90 ± 0.35 cm) and o*scdp3.10-4* (2.44 ± 0.31 cm) were significantly longer than those of ZH11 (1.88 ± 0.25 cm) (Fig. 5C, D). The lesion lengths of the *OsSPX3* CRISPR/Cas9 lines *osspx3-1* and *osspx3-2* were 1.27 ± 0.35 cm and 1.43 ± 0.21 cm, respectively, which were shorter than those of wild-type ZH11 (2.21 ± 0.36 cm) *(*Fig. 5E, F). We found that the lesion lengths of the *LOC_Os11g03820* CRISPR/Cas9 lines *cas9-1* (1.42 ± 0.16 cm), *cas9-2* (1.42 ± 0.13 cm) and *cas9-3* (1.51 ± 0.20 cm) were significantly longer than those of the wild-type KitaaKe (1.19 ± 0.12 cm) *(*Fig. 5G, H). A previous report showed that *OsDSR2* negatively regulates the response of rice to salt and drought stress and abscisic acid signaling [24]. After inoculation with HGA4, the lesion lengths of the *OsDSR2*-overexpressing lines OE-2-6 (1.79 ± 0.28 cm) and OE-13-4 (1.80 ± 0.31 cm) were shorter, while those of the OsDSR2-suppressed lines RNAi-5 (2.73 ± 0.43 cm) and RNAi-14 (2.48 ± 0.73 cm) were longer than that of ZH11 (2.34 ± 0.32 cm) *(*Fig. 5I, J). Taken together, the four randomly selected common DEGs were *DR* genes. *OsCDP3.10*, *LOC_Os11g03820* and *OsDSR2* were positively related to BLS resistance, while *OsSPX3* was negatively related to BLS resistance.

## Discussion

### Enriched *DR* genes by comparative transcriptomics analysis

BLS is an important rice quarantine disease in China [40], and the cultivation of disease-resistant varieties is needed for disease prevention. The discovery of the molecular mechanism of disease resistance is an important driving force for breeding disease-resistant varieties, both for *R* and *DR* genes [41]. In this study, we explored transcription profiles at the early (12 hpi) and late (3 dpi) stages after rice inoculation with Xoc by RNA-seq. Hundreds to thousands of DEGs were identified upon inoculation with a hypervirulent strain (HGA4) or a hypovirulent strain (RS105). The common DEGs were enriched for the two inoculated strains at 12 hpi, 3 dpi and both time points. The functional annotation of those common DEGs was performed by GO and KEGG analyses. The regulatory pathogenic mechanism in rice against different virulent Xoc strains is further understood. Consistent with the fact that RS105 is slightly less virulent than HGA4 and is a broadly used highly virulent Xoc strain [14], the number of DEGs identified in the two strains was approximately similar at both 12 hpi and 3 dpi (Figs. 2A and B and 3A). Importantly, we calculated the number of DEGs for HGA4 or RS105 at 12 hpi and 3 dpi independently and the number of common DEGs. Overall, there were fewer common DEGs than individual DEGs identified by HGA4 or RS105. The number of DEGs dramatically decreased from 8399 (common DEGs at 12 hpi) and 2313 (common DEGs at 3 dpi) to 1482 DEGs after integrated analysis of the common DEGs at both 12 hpi and 3 dpi (Fig. 4A). RNA-seq is very sensitive to various factors of developmental, environmental, biotic and abiotic stresses. RNA-seq analysis of the inoculation of two strains will be not only revealed the differential virulence involved genes, but also concentrated conserve *DRs* and reduced noise. These are also supported by recently published references for the inoculation of two fungi, *R. solani* YWK196 and YWK62, in maize [23]. Therefore, the *DR* genes were enriched by using comparative RNA-seq for inoculation with two closely related virulent strains.

### Revealed *DR* genes involved in the PTI and ETS responses in the rice-xoc interaction

During pathogen invasion, two-tiered immune responses, including early and rapid PTI and later but strong ETI, comprise the plant defense system. However, pathogens can deliver effectors that cause ETS to overcome PTI and ETI [2]. Two comparative transcriptional profiling studies have been performed to identify *DR* genes involved in BLS resistance [19, 20]. Different races of these Xoc strains were inoculated with hypervirulent or hypovirulent strains on a rice variety carrying the major *R* genes, and comparative analysis of the *DR* genes between incompatible and compatible rice-Xoc interactions was performed [19, 20]. In this study, we performed a comparative analysis of DEGs in response to two compatible Xoc strains and closely related virulent strains. Because ZH11 is susceptible to both HGA4 and RS105 and does not contain any identified *R* genes for BLS resistance, DEGs at 12 hpi and 3 dpi tended to be associated with the PTI response and ETS response, respectively. We found that more common genes were coexpressed with HGA4 and RS105 at 12 hpi than at 3 dpi (Figs. 2A and B and 3A). Notably, the fold change in common DEGs between HGA4 and RS105 was greater at 3 dpi than at 12 hpi (Fig. S2), and more putative TALE target genes were induced to be expressed at 3 dpi (Table 1), suggesting that those DEGs at 3 dpi may be related to the ETS response and determine the greater virulence of HGA4.

There was no significant difference in the bacterial populations between HGA4 and RS105 at 12 hpi (Fig. 1). The number of total DEGs and the relative expression levels of the common DEGs were similar between HGA4 and RS105. These results implied that the hypervirulent and hypovirulent Xoc strains triggered similar levels of PTI response. A comparison of the DEGs identified at 12 hpi and 3 dpi revealed that the number dramatically decreased from more than 9000 to less than 3000 for both HGA4 and RS105 (Figs. 2A and B and 3A). This finding is consistent with the findings obtained for maize-*R. solani* interactions [23]. Furthermore, in a comparative transcriptional analysis of *bls2*-mediated resistance to compatible Xoc strain (WT) and incompatible type III effectors deficiency Xoc strain (MT) interactions, there are 415 DEGs between WT and MT were identified at 12 hpi, while only 150 DEGs were found at 48 hpi [21]. Overall, PTI at 12 hpi seems to mediate a more complex signaling pathway than ETS at 3 dpi.

### Assistance to mine TALE target genes from DEGs at 3 dpi

TALEs secreted by the type III secretion system are pathogenic factors of Xoc. There are more than 28 TALEs in Xoc strains, but only a small number of TALEs have been studied [13, 31]. Tal2g in Xoc strain BLS256 can target the promoter of *OsSULRT3;6* to promote susceptibility [31]. Tal2h is a truncated TALE in BLS256 that interferes with *Xo1*-mediated resistance in the heirloom rice variety Carolina Gold through a direct protein‒protein interaction that is independent of its DNA binding activity [42]. Tal7 in Xoc strain RS105 activates the expression of the rice genes *LOC_Os09g29100* and *LOC_Os12g42970*, which suppresses *avrXa7*-*Xa7*-mediated ETI in rice [43]. Overexpression of Tal2a in BLS256 reduced virulence by targeting a ubiquitin carboxy-terminal hydrolase gene (*UCH*; *Os02g43760*) [44]. In HGA4, Tal2b and Tal2c target *OsF3H03g* and *OsF3H04g*, respectively, which encode rice 2-oxoglutarate-dependent dioxygenases that mediate SA metabolism [14, 15]. In addition to the above six TALEs, several other TALEs could be used to predict putative target genes by aligning the sequences of EBEs in the rice genome (Table 1). However, more than half of the TALEs in HGA4 could not be used to identify target genes [32, 33]. Here, we found that 10 putative TALE target genes were activated among 930 commonly upregulated DEGs at 3 dpi (Table 1). Five target genes of *OsHEN1*, *LOC_Os12g42970, LOC_Os01g31220, OsF3H03g* and *OsF3H04g*, were identified from 416 HGA4-specific upregulated genes whose expression was relatively high (Table 1). If other unidentical TALEs directly function as transcriptional regulators to activate target genes in the host, those targets will be identified from the common upregulated DEGs for common TALEs and from HGA4-specific upregulated DEGs for additional TALEs, such as Tal2d and Tal2e in HGA4.

### Functions of *DR* genes at 3 dpi

After comparative analysis, we identified 930 upregulated and 1383 downregulated common DEGs. However, it is still important to explore the function of each *DR* gene. According to the above discussion, the identified and identical TALE target genes were enriched in common upregulated *DR* genes, which are ordinarily regarded as *S* genes that negatively regulate rice immunity. Transcripts related to these *S* genes were coexpressed and enriched in DEGs at 3 dpi. We randomly validated the four candidates, including three upregulated and one downregulated *DR* gene. Surprisingly, *OsCDP3.10*, *LOC_Os11g03820* and *OsDSR2* positively regulated BLS resistance. Only *OsSPX3* negatively regulated resistance, as did the *S* genes (Fig. 5). Additionally, several *DR* genes involved in BLS resistance, such as positive regulators of *OsPGIP1* and *OsPSKR1* [12, 25, 45] and negative regulators of *OsNRAMP1* and *OsMAPK6/BLS1* [29, 46], were enriched among the common DEGs whose expression was upregulated at 3 dpi (Table 2). In conclusion, in addition to the DEGs associated with ETS genes, DEGs related to immune regulators were enriched at 3 dpi, indicating that a defense response or ETI still existed at 3 dpi.

Increasing evidence has demonstrated that bacterial pathogen effectors suppress host immunity by interfering with plant hormone production and signaling pathways [44]. *Pseudomonas syringae* produces coronatine, a toxin that mimics JA, which acts by antagonizing JA and SA signals to regulate crosstalk, resulting in impaired plant stomatal and apoplastic defenses [47]. Tal2b and Tal2c in Xoc HGA4 target *OsF3H03g* and OsF3H04g to mediate the hydroxylation of SA [14, 15, 48]. In this study, ET-responsive *OsERF1* and JA-responsive *OsJAZ13* were not induced at 12 hpi but were downregulated at 3 dpi (Fig. 4B and C). However, SA-related *OsPR1b* was not expressed at 12 hpi but was upregulated at 3 dpi (Fig. 4D). In addition to SA, JA and ET, microbial pathogens also target other plant hormone signaling pathways to regulate host immune responses. For example, *Pseudomonas syringae* T3SE AvrRpt2 antagonizes defenses during infection by increasing plant auxin levels [49]. In this study, we found that compared with those at 12 hpi, the number of auxin-related genes (Six genes were up-regulated at 12 hpi, but not at 3 dpi. Nine genes were down-regulated at 12 hpi, but not expressed or differentially expressed at 3 dpi.) and cytokinin-related genes (Eight genes were down-regulated at 12 hpi and were not expressed or differentially expressed at 3 dpi.) among the DEGs decreased at 3 dpi in the Xoc strain (Table S8). This indicates that during Xoc invasion, the production of virulence factors may interfere with auxin and cytokinin signal transduction and affect plant growth and development.

## Conclusion

In general, by performing comparative transcriptional profiling of ZH11 after inoculation with the hypervirulent strain HGA4 and the hypovirulent strain RS105, we identified 8399 and 2313 common DEGs at 12 hpi and 3 dpi, respectively. These *DR* genes are useful for exploring PTI and ETI/ETS, which are involved in both early and late defense responses. Furthermore, we identified four novel *DR* genes and validated their function in BLS resistance, which will be applied to improve disease resistance in rice in the future.

### Electronic supplementary material

Below is the link to the electronic supplementary material.

Supplementary Material 1. Additional file 1. Supplementary figures (**Figure S1**: GO analysis of the common DEGs at 12 h post inoculation with HGA4 and RS105; **Figure S2**: Histogram of the ratio of common DEGs at 3 dpi after inoculation with HGA4 and RS105; **Figure S3**: Expression levels determined by qRT‒PCR. **Figure S4**: Important KEGG pathways related to plant disease resistance mechanism.)

Supplementary Material 2. Additional file 2. Supplementary tables. **Table S1**: The listed of primers used in this study; **Table S2**: RNA sequencing data quality and mapping information; **Table S3**: Functional annotation of common DEGs at 12 hpi; **Table S4**: GO analysis of up-regulated common DEGs at 12 hpi; **Table S5**: GO analysis of down-regulated common DEGs at 12 hpi; **Table S6**: GO analysis of the up-regulated common DEGs at 3 dpi; **Table S7**: GO analysis of the down-regulated common DEGs at 3 dpi; **Table S8**: Plant hormone signaling pathway related genes. **Table S9**: The common DEGs at 3 dpi with HGA4 and RS105.

## Data Availability

All data that support the findings in this study are available in this article and its supplementary files (Supplementary Table S1-S8; Fig. S1, S2, S3, S4). Sequence data from this study can be found on the Rice Genome Annotation Project website (http://rice.plantbiology.msu.edu/, accessed on 10 September 2021) and NCBI (https://www.ncbi.nlm.nih.gov/, accessed on 17 June 2022) under the following accession number: *OsCDP3.10* (*LOC_Os03g57960*), *OsSPX3* (*LOC_Os10g25310*), *LOC_Os11g03820*, *OsDSR2* (*LOC_Os01g62200*), *LOC_Os08g07080*, *LOC_Os12g08760*, *LOC_Os06g08580*, *LOC_Os01g08020*, *LOC_Os08g38170*, *LOC_Os01g65140*, *OsDR8* (*LOC_Os07g34570*), *LOC_Os04g14690*, *LOC_Os03g08580*. Raw sequence reads of transcriptome sequencing for ZH11, HGA4_12 h, HGA4_3 d, RS105_12 h and RS105_3 d were performed in this study and deposited to SRA to achieve the accession number PRJNA1033788.

## Abbreviations

BLS: Bacterial leaf streak
BLSB: Banded leaf and sheath blight
DEGs: Differentially expressed genes
Dpi: days post inoculation
DR: Defense-responsive
EBEs: Effector-binding elements
ET: Ethylene
ETI: Effector triggered immunity
ETS: Effector triggered susceptibility
GO: Gene Ontology
Hpi: Hours post inoculation
HR: Hypersensitive response
JA: Jasmonic acid
KEGG: Kyoto Encyclopedia of Genes and Genomes
MAPK: Mitogen-activated protein kinase
NLRs: Nucleotide-binding domain and leucine-rich repeat receptors
PAMPs: Pathogen associated molecular patterns
PRRs: Pattern recognition receptors
PTI: PAMPs triggered immunity
R: Resistance
RLKs: Receptor-like kinases
RLPs: Receptor-like proteins
ROS: Reactive oxygen species
RVDs: Repeat-variable diresidues
S: Susceptibility
SA: Salicylic acid
TALE: Transcription activator like effector
TF: Transcription factor
T3SS: Type III secretion system
Xoc: *Xanthomonas oryzae* pv. *oryzicola*
ZH11: Zhonghua 11
