# Supplementary material for: Comparative transcriptomic profiling of the two-stage response of rice to Xanthomonas oryzae pv. oryzicola interaction with two different pathogenic strains
Source: BMC Plant Biol. 2024 Apr 29;24:347. doi: 10.1186/s12870-024-05060-1 (PMC11057074; doi:10.1186/s12870-024-05060-1)
Supplement: Supplementary file 1 — Supplementary Material 1. Additional file 1. Supplementary figures (Figure S1: GO analysis of the common DEGs at 12 h post inoculation with HGA4 and RS105; Figure S2: Histogram of the ratio of common DEGs at 3 dpi after inoculation with HGA4 and RS105; Figure S3: Expression levels determined by qRT‒PCR. Figure S4: Important KEGG pathways related to plant disease resistance mechanism.) [file 12870_2024_5060_MOESM1_ESM.docx]

**Comparative transcriptomic profiling of the two-stage response of rice to *Xanthomonas oryzae* pv. *oryzicola* interaction with two different pathogenic strains**

Yunya Bi, Yue Yu, Shuaige Mao, Tao Wu, Tao Wang, Ying Zhou, Kabin Xie, Hua Zhang, Li Liu, Zhaohui Chu

*Correspondence: Zhaohui Chu (ORCID: 0000-0001-8320-7872) zchu77@whu.edu.cn; Li Liu liuli2020@hubu.edu.cn

^†^These authors contributed equally to this work.


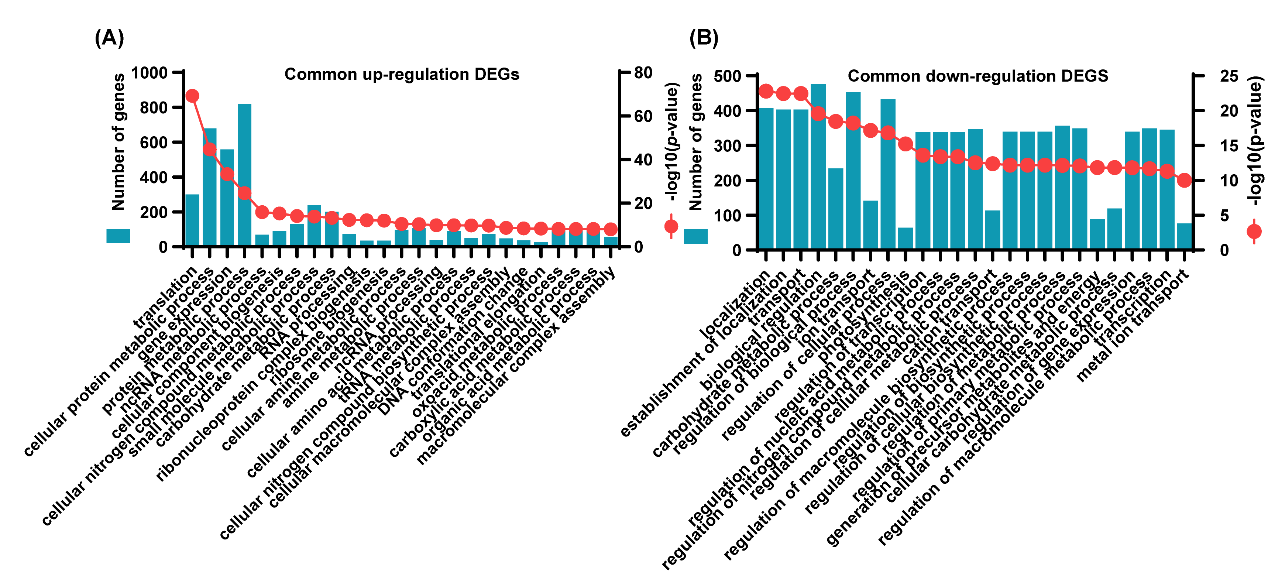


**Figure S1:** GO analysis of the common DEGs at 12 hours post inoculation with HGA4 and RS105. Biological process analysis of the common DEGs for upregulated genes (A) and downregulated genes (B).


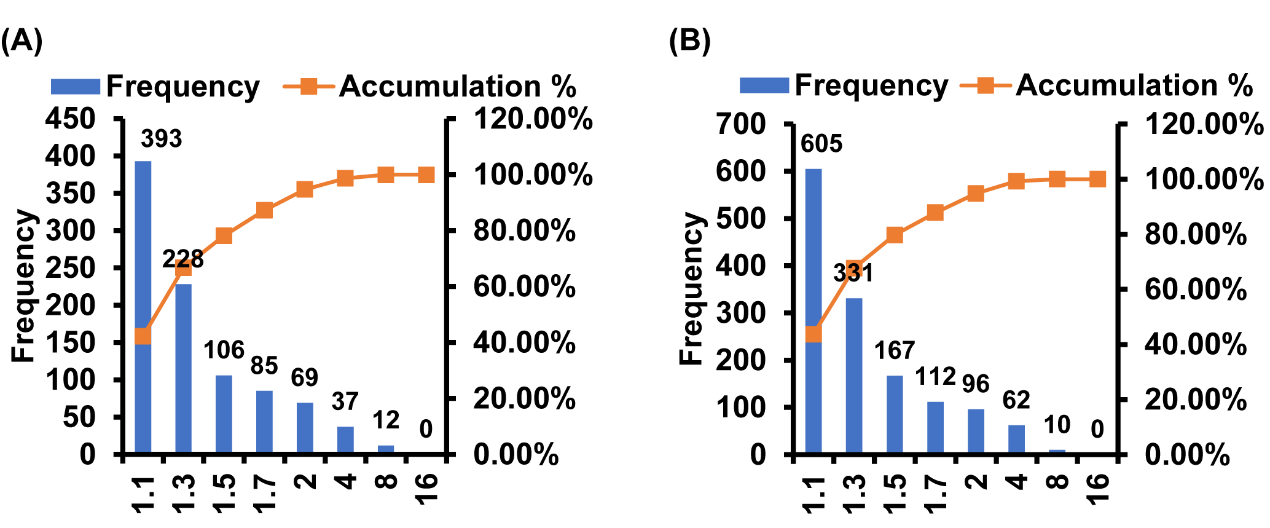


**Figure S2:** Histogram of the ratio of common DEGs at 3 dpi with HGA4 and RS105. Histogram of the ratio of up-regulated common DEGs (A) to down-regulated common DEGs (B) at 3 dpi with HGA4 and RS105.


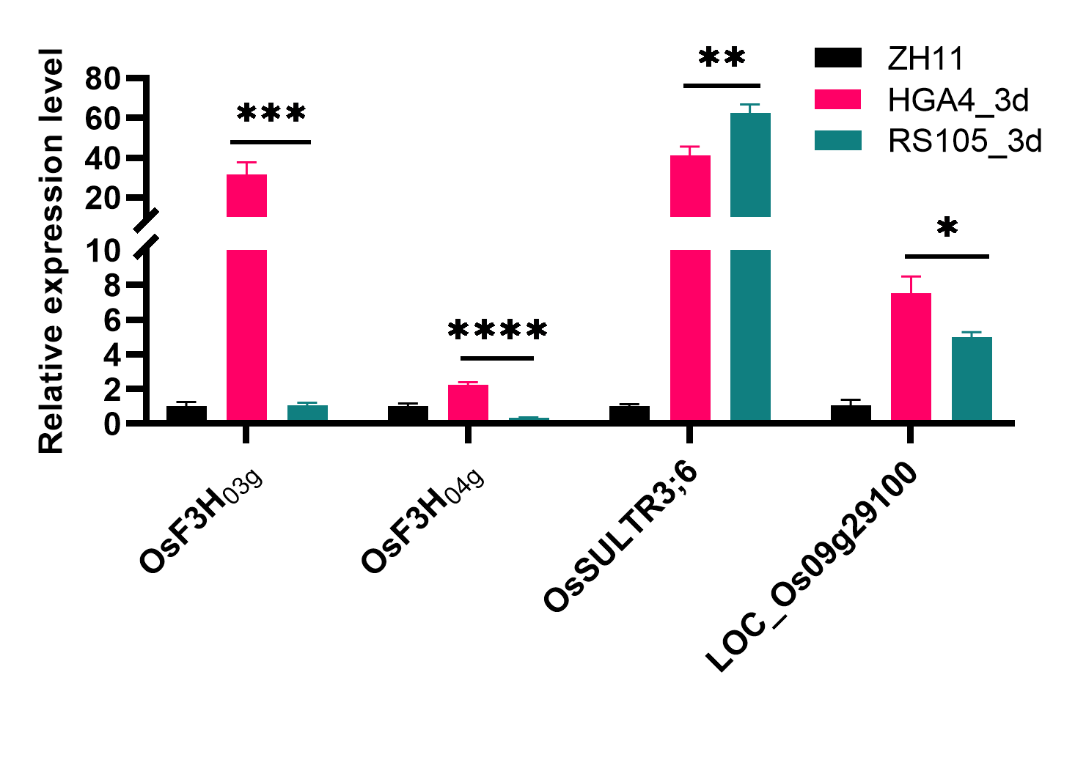


**Figure S3:** Expression levels determined by qRT‒PCR. qRT-PCR was used to verify the expression of DEGs at 3 dpi with HGA4 and RS105. Unpaired *t* test, **P*≤0.05, ** *P* ≤ 0.01, *** *P*≤ 0.001, **** *P*≤ 0.0001.


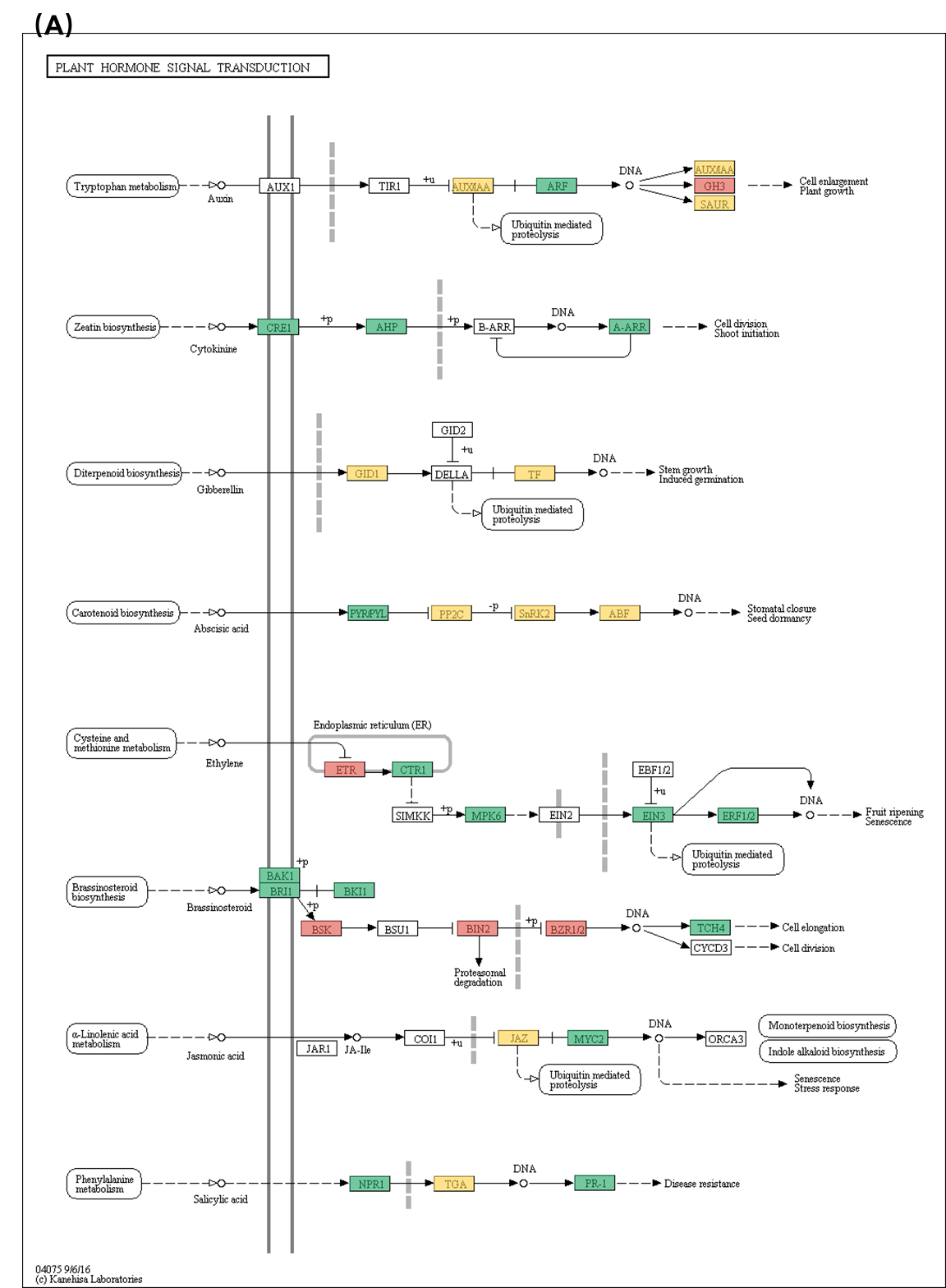


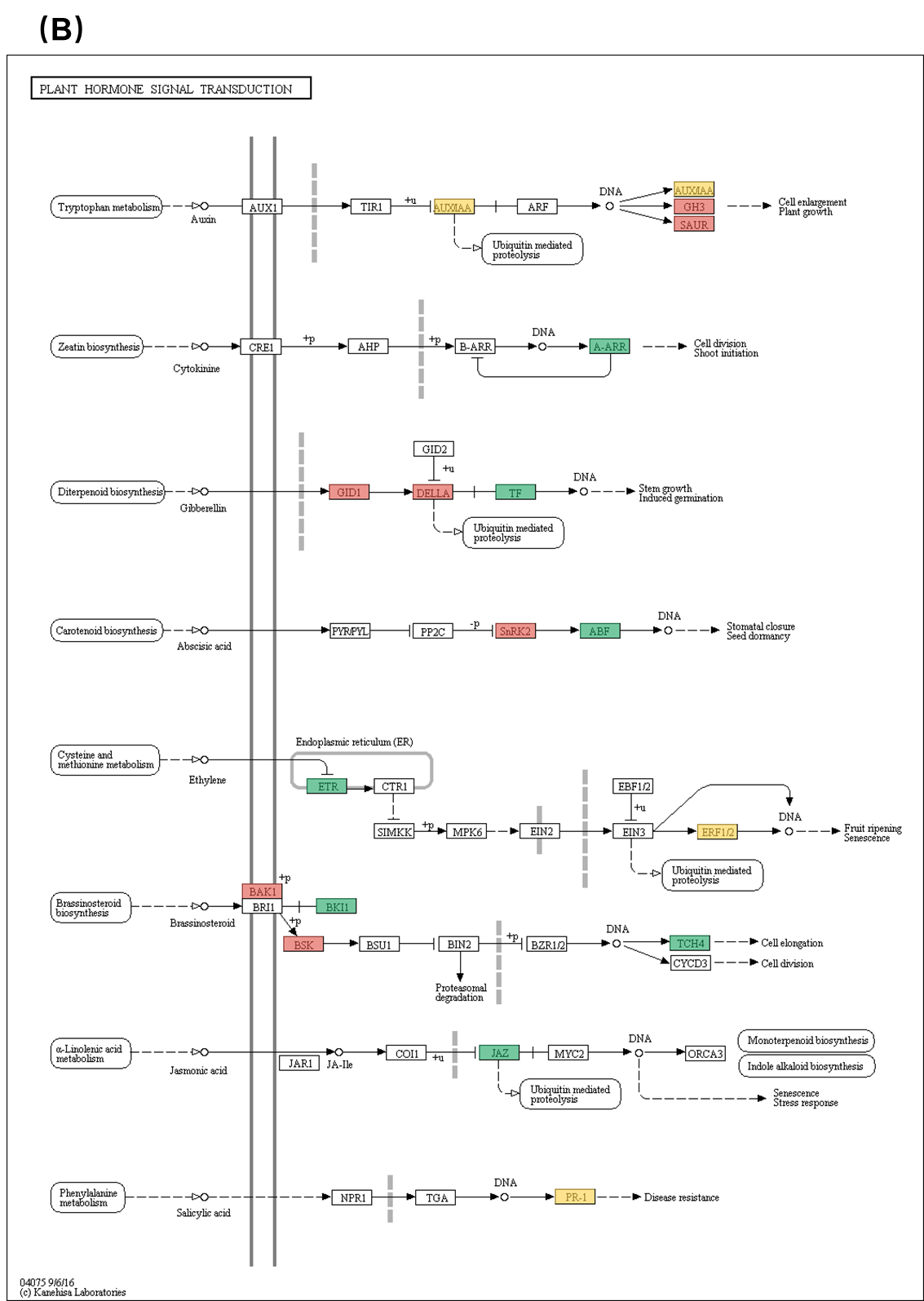


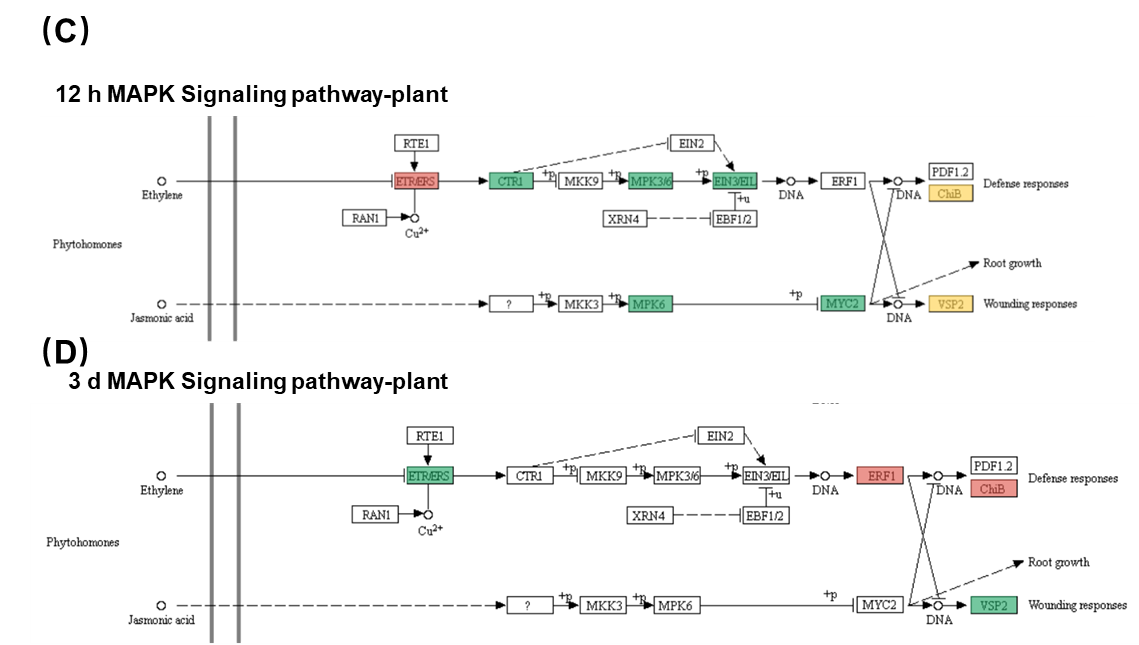


**Figure S4:** Important KEGG pathways related to plant disease resistance mechanism. The plant hormone signal transduction of 12 hpi (A) and 3 dpi (B) and the MAPK signaling pathway-plant of 12 hpi (C) and 3 dpi (D). Red: up-regulated; Green: down-regulated; Yellow: both up-regulated and down-regulated.
